# The Challenge of Applying and Undertaking Research in Female Sport

**DOI:** 10.1186/s40798-019-0224-x

**Published:** 2019-12-12

**Authors:** Stacey Emmonds, Omar Heyward, Ben Jones

**Affiliations:** 1 Institute for Sport, Physical Activity and Leisure, Leeds Beckett University, Leeds, UK; 2 England Performance Unit, Rugby Football League, Red Hall, Leeds, UK; 3 Rugby Football Union, Twickenham, London, UK; 4 Leeds Rhinos Rugby League Club, Leeds, UK; 5 School of Science and Technology, University of New England, Armidale, New South Wales Australia; 6 Division of Exercise Science and Sports Medicine, Department of Human Biology, Faculty of Health Sciences, University of Cape Town and the Sports Science Institute of South Africa, Cape Town, South Africa

## Abstract

In recent years there has been an exponential rise in the professionalism and success of female sports. Practitioners (e.g., sport science professionals) aim to apply evidence-informed approaches to optimise athlete performance and well-being. Evidence-informed practices should be derived from research literature. Given the lack of research on elite female athletes, this is challenging at present. This limits the ability to adopt an evidence-informed approach when working in female sports, and as such, we are likely failing to maximize the performance potential of female athletes. This article discusses the challenges of applying an evidence base derived from male athletes to female athletes. A conceptual framework is presented, which depicts the need to question the current (male) evidence base due to the differences of the “female athlete” and the “female sporting environment,” which pose a number of challenges for practitioners working in the field. Until a comparable applied sport science research evidence base is established in female athletes, evidence-informed approaches will remain a challenge for those working in female sport.

## Key Points

- There is currently a lack of sport science and sport medicine research conducted on elite female athletes, making it challenging to develop an evidence-informed approach to practice.
- Applying evidence developed in male athletes to female athletes may be erroneous.
- This article highlights the challenges of applying evidence derived from male athletes and applying it to female athletes and female sporting contexts. It provides considerations of how to apply research to female sport, considering the female athlete and the female sporting environment.

## Introduction

In recent years there has been an exponential rise in the professionalism and profile of female sports [1]. Women’s professional soccer, rugby, and netball leagues now exist in a number of countries. While still acknowledging the disparity in opportunities, salaries, and media exposure between elite male and female athletes [1], the increased professionalism has afforded female athletes the opportunity to train full time and also access professional sports coaching, sport science, and sports medicine support to help maximize performance potential.

Practitioners (e.g., sport science professionals) aim to apply evidence-informed approaches to accomplish the goal of optimal athlete performance and well-being. Evidence-informed practice is the application of research findings to the real world [2]. The challenges of applying research to practice in sport have been highlighted in the literature (i.e. the challenge of translating science to the context) [2]. This is even more challenging when working with female cohorts. While in recent years more female participants are included within the research literature, these studies typically involve recreational athletes [3], and as such high-performance female athletes are typically underrepresented in the “sports performance” literature. This limits the ability to adopt and apply an evidence-informed approach when working with elite female athletes and as such may mean that we are failing to maximize the performance potential of this cohort.

The purpose of this article is therefore to highlight the challenges of applying evidence developed in a male cohort to a female cohort. Of note, this article will not discuss gender as it is outside the scope of this editorial. The article will provide considerations, supporting the application of evidence into practice, and also support future translational research for female sport.

## Considerations when Applying Sports Performance Research to Female Athletes

Scientific research aims to investigate the effects of *independent variables* (e.g., age, maturation status, a training intervention) on *dependent variables* (e.g., sprint performance). In sports science disciplines, sex should be controlled given the different biological attributes [4]. However, sport science practices (e.g., training and recovery protocols, nutritional strategies, injury prevention interventions) in female sport are often underpinned by research conducted in male athletes, given the limited representation of female athletes in the sports performance literature. The underrepresentation is highlighted by a search of “injury” and “rugby” and “female” in the last 10 years of retrieving 196 articles, whereas the same search, replacing “female” for “male,” retrieved 602 articles. A similar trend was also observed for “soccer match demands,” with 13 and 102 articles retrieved for females and males, respectively (Scopus, 19 July 2019). These corroborate recent findings showing that only 35% of participants are female in studies published in the *British Journal of Sports Medicine* [5]. The application of evidence derived in male athletes to female athletes is a concern given the known biological differences between the sexes.

Developing an applied sports performance evidence base in female sport is also challenging, given the logistical and methodological context [6]. Fluctuations in hormone concentrations at different stages of the menstrual cycle may influence performance [8]. This is in addition to the different biomechanical profiles of female athletes in comparison to male athletes [6]. These factors may partially account for the lack of efficacy and effectiveness of interventions [7] when applying findings from sports performance research conducted in male athletes. For example, it is known that estrogen concentrations fluctuate throughout the menstrual cycle and estrogen has measurable effects on muscle function and tendon and ligament strength [8]. Estrogen and relaxin concentrations have been reported to peak during the luteal phase of the menstrual cycle, potentially increasing anterior cruciate ligament (ACL) injury risk [9]. Similarly, fluctuations in estrogen and progesterone concentrations during different stages of the menstrual cycle may affect temperature regulation, central nervous system fatigue, substrate metabolism, and overall exercise performance [7]. Therefore, female athletes may require different performance, nutritional, recovery, and injury prevention strategies in comparison to male athletes.

Contextual factors may also influence the effectiveness and application of sports science interventions in practice. Contextual factors include competition structure, finance allocated to tournaments, access to facilities, or access to expert staff, for example. Sports science and medical provision (e.g., strength and conditioning, physiotherapy, team doctor, nutrition) are often limited for female athletes in comparison to males and must be considered when trying to apply research to practice. For example, the success of a training or injury prevention intervention is not solely determined by the efficacy of the intervention, but it is also influenced by multiple interrelated contextual factors within the target group and in the community [10]. Specifically, return to play guidelines in sport (i.e., soccer, rugby) are the same for both sexes, yet female athletes have been reported to have higher concussion rates [11] and present different concussion symptoms [12]. When considering the return to play from injury, contextual challenges, such as access to appropriate qualified support staff (e.g., physiotherapist, sports science support), in addition to the previously identified biological differences, should be considered when supporting female athletes.

## Developing and Applying Sports Science Evidence for Female Athletes

Current sports performance and player well-being strategies in female sport are often underpinned by evidence derived from male athletes or male talent development environments. While there are some good practices that can be derived from a male context, in some instances we may be failing to consider the requirements of the female athlete as highlighted above. When aiming to either develop applied sport science practices, adopt an evidence-informed approach, or undertake future research, the first step is to appraise and evaluate the current available evidence. Acknowledging that limited research studies have investigated female athlete cohorts in comparison to male athletes, this may lead to simply identifying the “best available evidence.” For the practitioner, this may mean that the evidence is useful to support decision-making or indeed the findings may not be suitable to translate into practice, due to inherent differences (e.g., talent development systems in male youth soccer *vs.* female youth soccer).

**Fig. 1:**
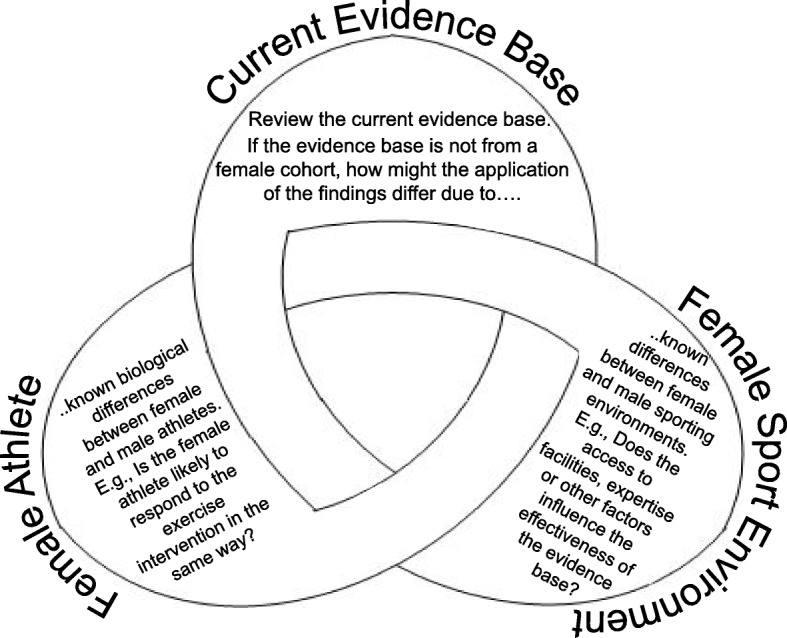
Considerations required when developing an evidence-based approach to practice in female sport

In Fig. 1 (adapted from Hanson et al. [13]), we propose the considerations required when aiming to develop an evidence-based approach to practice in female sport. The figure highlights how it is important that the current evidence base is evaluated against (a) *the female athlete* and (b) *the female sporting environment*, in addition to the typical scientific scrutiny applied to published research literature. This can be used to both apply the current evidence into policy and practice and indeed conceive future research projects specific to the needs of the female athletes, which has direct translation into practice.

For example, there is a strong body of research evaluating the match demands of male rugby league [14], but at present limited research exists evaluating the match demands of female rugby league. Following the considerations presented in Fig. 1, by establishing that the *female athlete* (e.g., rugby league player) is different to the male rugby league player (e.g., male vs. female rugby league players 20-m speed; 3.66 ± 0.26 vs. 3.09 ± 0.12 s [15, 16]), it is unlikely that the match demands research from male rugby league players can be applied to female cohorts. Furthermore, rugby league is professional in England and Australia for elite males and amateur and semiprofessional for elite females; thus when considering the *female sporting environment* and it’s context, this further corroborates the conclusion that match demands research form male cohorts have limited application to female cohorts.

Acknowledging that the effective translation of research findings is not solely determined by the efficacy of the intervention [13], there is a clear need to consider the “context” and “environment” of female sport, acknowledging that what occurs in the male game may not be most appropriate for the environment of females. For example, despite the increased professionalism of female sport, factors, such as insufficient training time and lack of resources and equipment in comparison to male athletes, may limit the ability of practitioners to apply such intervention-based evidence to practice. For example, within this context, professional medical staff may not be present at all training session, or qualified sports science/strength and conditioning practitioners, given the limited funding at present in some female sports.

The application of established research models, considering the female athlete within context, is likely a useful starting point. Bishop [17] provides a framework for undertaking applied research, progressing from “descriptive research” (e.g., what do they do) to “implementation studies in real sporting settings” (e.g., can we improve current practice). Jones et al. [18] also proposed a research model, emphasizing the need to co-construct research questions with policy-makers and practitioners to increase the *usefulness* and adoption of the research findings into practice. Adopting such approaches to research as described by Bishop [17] and Jones et al. [18] with considerations for the needs of the female athlete and the context of female sport will increase our understanding of the current context (i.e., physical qualities of players, match characteristics, recovery profiles, etc.), which, for a number of reasons discussed above, may be different to that in male athletes within the same sport. These studies are arguably more valuable at present than more advanced scientific studies (e.g., laboratory-based randomized crossover design studies). The challenge for the researcher is that this may be seen by journal editors and academic hierarchies as lacking “originality,” given the potential methodological repetition of male research in a female cohort. While this may be true for the advancement of scientific methodologies, it is an essential first step in the research process to understand the context of female sport and the female athlete. Even within male cohorts, a call for research reproducibility has been made [19]; thus the need to replicate studies from male cohorts in female cohorts is required.

## Conclusion

In summary, all stakeholders need to be cognizant of sexual dimorphism and the disparity in the current sports science literature and consequent challenges of adopting an evidence-informed approach to practice for female athletes. When applying and undertaking research in female sport, the first step is to appraise and evaluate the current available evidence, with consideration for both the *female athlete* and the *female sporting environment*. Considering the athlete and the environment allows the researcher and practitioner to consider potential differences to published literature in male cohorts. Due to the dearth in female-specific sport science literature, in most cases there is a clear need to start with descriptive research to understand the current level of performance within female elite sport. Once this is achieved, the next challenge will be exploring in the influence of female physiology and the contextual factors which may limit the effectiveness of interventions with high efficacy. Only when this disparity in applied sport science research is addressed will the full potential of adopting an evidence-informed approach be possible in female sport.

## Data Availability

Not applicable

## Abbreviations

ACL: Anterior cruciate ligament
